# The Influence of Mesoscopic Surface Structure on the Electrocatalytic Selectivity of CO_2_ Reduction with UHV-Prepared Cu(111) Single Crystals

**DOI:** 10.1021/acsenergylett.3c02693

**Published:** 2024-01-29

**Authors:** Khanh-Ly C. Nguyen, Jared P. Bruce, Aram Yoon, Juan J. Navarro, Fabian Scholten, Felix Landwehr, Clara Rettenmaier, Markus Heyde, Beatriz Roldan Cuenya

**Affiliations:** Department of Interface Science, Fritz Haber Institute of the Max Planck Society, Faradayweg 4-6, Berlin 14195, Germany

## Abstract

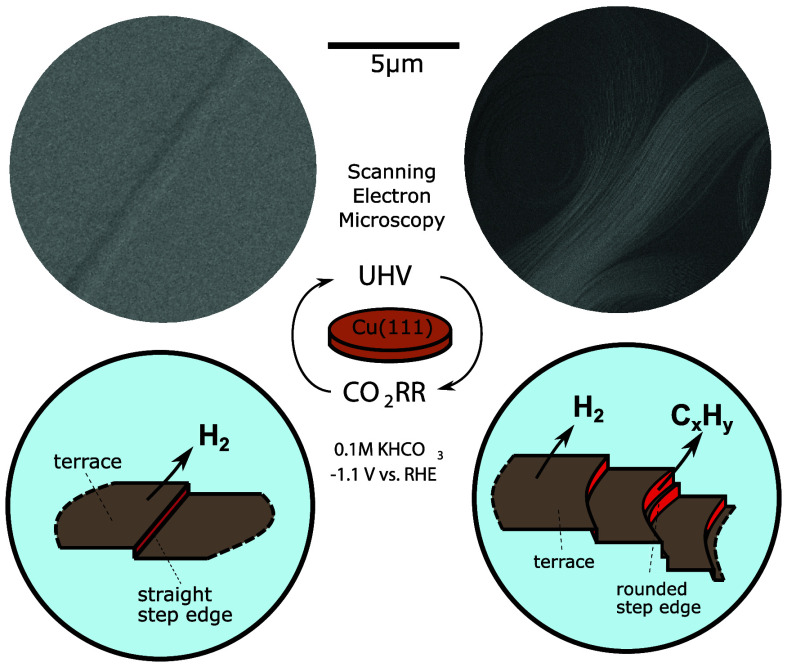

The key role of morphological defects (e.g., irregular steps and dislocations) on the selectivity of model Cu catalysts for the electrocatalytic reduction of CO_2_ (CO_2_RR) is illustrated here. Cu(111) single-crystal surfaces prepared under ultrahigh vacuum (UHV) conditions and presenting similar chemical and local microscopic surface features were found to display different product selectivity during the CO_2_RR. In particular, changes in selectivity from hydrogen-dominant to hydrocarbon-dominant product distributions were observed based on the number of CO_2_RR electrolysis pretreatment cycles performed prior to a subsequent UHV surface regeneration treatment, which lead to surfaces with seemingly identical chemical composition and local crystallographic structure. However, significant mesostructural changes were observed through a micron-scale microscopic analysis, including a higher density of irregular steps on the samples producing hydrocarbons. Thus, our findings highlight that step edges are key for C–C coupling in the CO_2_RR and that not only atomistic but also mesoscale characterization of electrocatalytic materials is needed in order to comprehend complex selectivity trends.

Electrocatalytic reduction of CO_2_ (CO_2_RR) to higher-order hydrocarbons has been proposed as one of the many tools available to help mitigate the effects of anthropogenic climate change and create a carbon-neutral energy cycle.^1^ The only pure metal that is capable of electrocatalytically reducing CO_2_ to C_2+_ hydrocarbons and alcohols with significant yields is copper (Cu). However, Cu suffers from overall low selectivity toward these products.^2^ Since the CO_2_RR is a complex proton-coupled multielectron transfer reaction, a wide range of products ranging from C_1_ products (carbon monoxide (CO), formate (HCOO^–^), methane (CH_4_)) to high-value C_2+_ products (e.g., ethylene (C_2_H_4_), ethanol (C_2_H_5_OH), and 1-propanol (C_3_H_7_OH)) can be obtained.^3−9^ To tailor the reaction pathway toward C_2+_ products, many studies have focused on tuning the intrinsic catalytic performance of Cu.^9−13^ In oxide-derived copper catalysts, the increased selectivity toward C_2+_ products is thought to be maintained by either the modified rough surface structure left behind after the electrochemical Cu_*x*_O reduction^9,10^ or by the partial stabilization of (sub)surface oxides or subsurface oxygen during reducing conditions.^11^ On nanostructured electrodes, the surface morphology plays an important role in manipulating the selectivity depending on the size and shape of the nanocrystals.^12^ Nanomaterials have higher amounts of undercoordinated sites available or preferential facets exposed, that have been correlated with specific activity and selectivity trends.^13,14^ Although high yields for C_2+_ products at reasonable current densities were achieved in recent years,^15−17^ a fundamental understanding of the nature of the catalytic active sites still remains elusive.

Experimental studies on Cu(hkl) single-crystal surfaces^18,19^ as well as theoretical calculations^20−22^ aim to elucidate the unique electrocatalytic properties of metallic copper. Nonetheless, theoretical studies thus far have relied on perfect (flat, atomically ordered, defect free) model surfaces and have largely neglected possible structural changes taking place at the electrode surface during CO_2_RR or even during common experimental surface pretreatments.^23^ In fact, to date most related experimental literature has investigated electropolished single-crystal surfaces,^9,19,24,25^ which are very rough and defective, in contrast to the long-range ordered pristine surfaces considered in theory.^26,27^ Only recently, theoretical attempts have been made to classify C_2+_ active sites on roughened Cu electrodes.^28^

In the present contribution, atomically flat ultrahigh vacuum (UHV)-prepared Cu single-crystal surfaces will function as model catalysts to enable a better connection between experimental work and theoretical calculations. Recently, we showed that atomically flat UHV-prepared copper surfaces favor the Hydrogen Evolution Reaction (HER) over the CO_2_RR.^23^ Only by introducing defects and high index sites by harsh treatments such as chemical etching, product distributions involving hydrocarbons were observed. The nature and identity of these CO_2_RR active sites are, however, still an open question.

While the majority of the prior studies has focused on the nanoscale range in order to attribute active sites to the overall intrinsic selectivity, it is important to point out that catalytic processes may also be influenced by length scales beyond atomic ranges. For instance, on well-defined mesostructured inverse opal Au, Ag, and Cu electrodes with controlled thickness, mesostructuring could be used for tuning the selectivity in CO_2_RR.^29−31^ In particular, the transport limitations occurring in such mesoporous structures were used to tune the selectivity toward CO_2_RR products versus H_2_. Recent works have also pointed out the important role of the electrode–electrolyte interface, including the formation of Cu hydroxide/carbonate species^32^ or through pH-dependent modifications of CO binding.^33^ Further works on polycrystalline Au surfaces have shown an increased CO_2_RR activity at grain boundaries.^34,35^ Grain boundaries serve as accumulation sites for dislocations and under-coordinated sites, proving that larger length scales (certainly beyond the atomistic calculations currently widely available) must be included in the investigation of electrocatalytic processes to fully understand the overall selectivity and activity trends of real materials.

In this study, we characterize UHV-prepared Cu(111) surfaces, exposed to a different number of CO_2_RR electrolysis and subsequent surface regeneration pretreatments, from the atomic to the micrometer scale. We have introduced minimal changes in the surface structure that were found to still drive significant selectivity changes in CO_2_RR. Here we show that for well-ordered Cu(111) surfaces, the product selectivity varies drastically from favoring HER to high hydrocarbon yields depending on the mesoscopic structure of the surface, in particular, the density and orientation of morphological irregularities such as atomic steps or step bunches. Thus, our study contributes to our understanding of the nature of the active motifs in CO_2_RR.

In our work, we ran multiple CO_2_RR cycles on the same UHV-prepared Cu(111) single crystal. Prior to each electrocatalytic reaction, the Cu(111) surface is regenerated by a UHV cleaning pretreatment described in the Experimental section (see Supporting Information). We used Low Energy Electron Diffraction (LEED) and Auger Electron Spectroscopy (AES) to characterize the surface before each CO_2_RR (Figure 1a–d). After the UHV preparation, the Cu(111) single crystal is mounted *ex situ* in an in-house fabricated sample holder,^36^ and CO_2_RR was measured at −1.1 V vs RHE in 0.1 M KHCO_3_. Since our work focuses on the study of the active sites for hydrocarbon production in CO_2_RR, a potential was chosen based on the work of Huang et. al,^25^ who reported the highest amount of hydrocarbon production at −1.1 V vs RHE for electropolished Cu(111). There, we found after 1 h of the CO_2_RR two different product distributions despite the same surface preparation process on the same single crystal, as can be clearly seen in Figure 2a. One product distribution is in agreement with the one we previously reported on atomically flat UHV-prepared single crystals, namely, H_2_ production.^23^ The HER is favored over CO_2_RR on the surface described in Figure 1a,c with 88% Faradaic Efficiency (FE) for hydrogen and only 3% for gaseous hydrocarbon products. We have termed this specific product distribution as ‘hydrogen product distribution (H_2_PD)’ for simplicity. The H_2_PD can be obtained for various applied potentials as seen in Figure S1. The second observed product distribution consists of a significantly higher amount of hydrocarbons (53%) with only 40% H_2_. The total FE for hydrocarbons is mainly caused by the high increase of methane production from 1% to 40%. Ethylene production increases from <1% to 12%, whereas the FE for CO remains almost negligible with 1%. We have termed this product distribution, observed on the surface described in Figure 1b,d, as ‘hydrocarbon product distribution (HCPD)’ in the following text.

**Figure 1:**
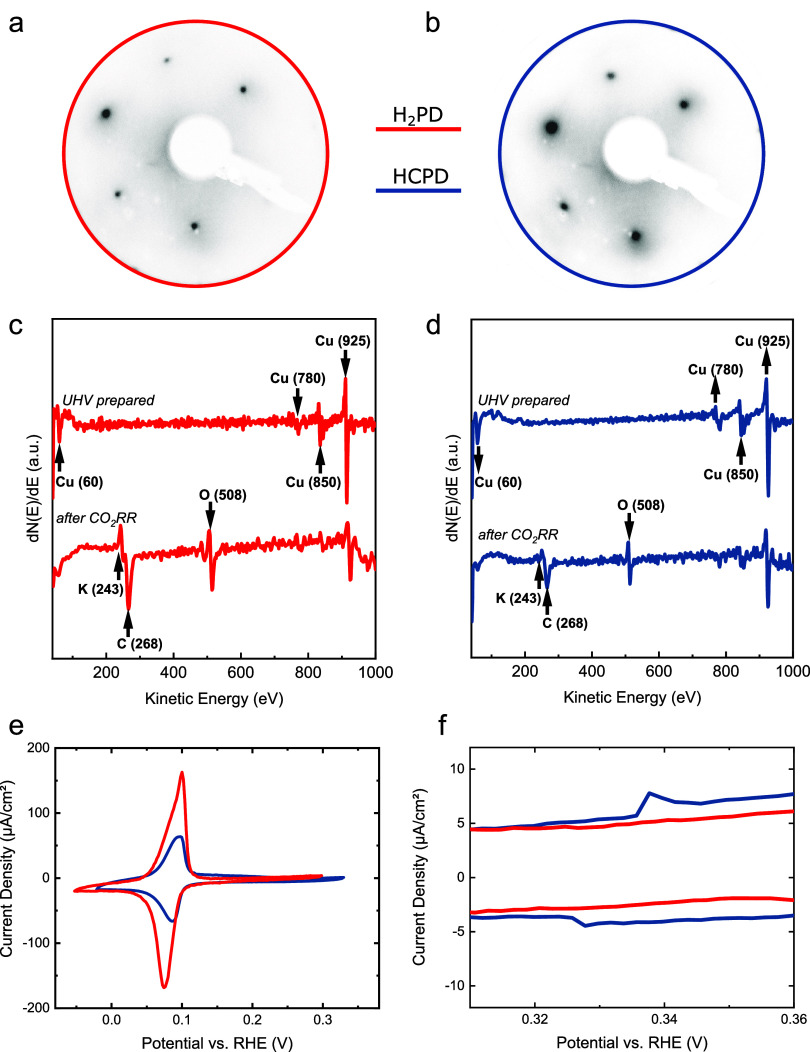
Surface characterization of a hydrogen producing (H_2_PD) (a, c) and a hydrocarbon producing (HCPD) (b, d) UHV-prepared Cu(111) single-crystal surface. LEED patterns of the (a) H_2_PD and (b) HCPD Cu(111) as-prepared surfaces. LEED was taken at *E* = 114 V. AES data of the same two as-prepared surfaces are shown in (c, d), correspondingly. CV scans of the UHV-prepared H_2_PD and HCPD surface (e) for the OH-adsorption/desorption region and (f) for the additional surface feature appearing for HCPD. Scan rate is 50 mV/s, and electrolyte is Ar-saturated 0.1 M NaOH. The CVs were measured without air exposure under Ar atmosphere before CO_2_RR.

**Figure 2:**
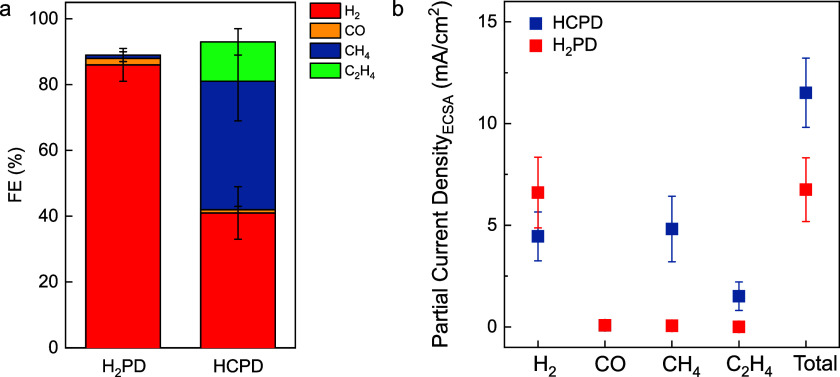
(a) Faradaic efficiency and (b) ECSA-normalized current densities after 1 h of CO_2_RR at −1.1 V vs RHE in 0.1 M KHCO_3_ of the H_2_PD and HCPD Cu(111) surfaces.

The hexagonal reciprocal lattice of the Cu(111) structure is shown in Figure 1a,b. The sharp, round spots in the LEED pattern indicate high crystallinity and an atomically well-defined structure. At first sight, the LEED patterns in Figure 1a,b display a similar general surface structure, as demonstrated by the identical number, shape, and symmetry of the spots. However, smaller differences can be detected in the spot size and background. The HCPD surface depicts larger spot sizes and increased background scattering as compared to the H_2_PD surface, which is indicative of shorter terraces in the former.^37^

The AES spectra for the UHV-prepared surfaces in Figure 1c,d show the characteristic Cu MVV peak at *E*_k_ = 60 eV and Cu LMM peaks at *E*_k_ = 780, 850, and 925 eV. Within the resolution of the AES method (0.1 at %), no contamination related to carbon or oxygen is observed on the as-prepared samples, highlighting the cleanliness of these surfaces. After CO_2_RR as seen in Figure 1c,d, additional carbon (C, KLL peak at *E*_k_ = 288 eV), oxygen (O, KLL peak at *E*_k_ = 508 eV), and potassium (K, LMM peak at *E*_k_ = 243 eV) signals were detected via AES. These species are attributed to electrolyte residues as well as to sample exposure to air during the transfer from the electrochemical cell to the AES analysis chamber. Moreover, there are no signs of contamination from the experimental setup.

In addition, cyclic voltammetry (CV) scans were performed on both as-prepared H_2_PD and HCPD surfaces in a quasi-in situ EC cell under an Ar atmosphere to probe for differences in the electrochemical behavior due to the different initial structures suggested by LEED. The samples were transferred directly from UHV to an Ar atmosphere without air exposure. In Figure 1e, we see the reversible OH-adsorption feature at 0.1 V vs RHE, which has been assigned to the adsorption of OH^–^ ions on {111} terraces.^38^ Comparing the H_2_PD and HCPD surfaces, we see that the OH^–^ adsorption and desorption is more pronounced for the H_2_PD surface than for the HCPD surface. The height of the OH-adsorption peak of the H_2_PD is more than twice times higher (∼165 μA/cm^2^) than for the HCPD surface (∼65 μA/cm^2^). This indicates a larger number of surface sites for OH adsorption on the H_2_PD surface. In addition, Figure 1f unveils an additional peak at 0.33 V vs RHE in the HCPD sample. In the literature, this feature is assigned to the OH adsorption on Cu(110) surfaces.^39^ This hints that the successive CO_2_RR cycles followed by a UHV sample regeneration treatment lead to a partial reconstruction of the Cu(111) surface toward domains with Cu(110) surface features.

Although the recovery of the surface via UHV treatment was expected, the small changes in the LEED spectra (Figure 1a,b) and the CVs (Figure 1e,f) after repeated CO_2_RR treatments suggest an irreversible surface restructuring process.

Not only does the product selectivity vary on UHV-prepared Cu(111) surfaces but also the activity, as displayed in Figure 2b. The respective current densities for the H_2_PD and HCPD Cu(111) surfaces are normalized to the electrochemical surface area (ECSA). The details of the ECSA calculation are found in Figure S2. Comparing the ECSA-normalized partial current densities for the hydrogen and hydrocarbon product distributions, one observes that the partial current density for H_2_ stays similar for the HCPD and the H_2_PD surface. We see that the overall increase in activity is caused by the increase of the partial current densities for the hydrocarbon products such as methane and ethylene. It should be noted that the ex situ AES post mortem (after CO_2_RR) chemical analysis of both surfaces reveals no clear differences within the resolution of this technique, Figure 1c,d.

To gain further insight into how possible surface restructuring taking place during the CO_2_RR affects the selectivity, we have collected data as a function of the reaction time and after different reaction cycles, each of them separated by a regeneration of the single crystal in UHV. To clarify here, the authors define a CO_2_RR cycle as the UHV preparation and subsequent CO_2_RR measurement, e.g., a surface after its fifth CO_2_RR cycle has passed five times through the cycle of UHV preparation and subsequent CO_2_RR measurement. Thus, Figure 3 demonstrates how the history of the Cu(111) single crystal influences the obtained product distribution. As the Cu(111) single crystal passes through several CO_2_RR cycles, the selectivity changes from an H_2_PD to an HCPD. In its first CO_2_RR run, a UHV-prepared Cu(111) single crystal produces mainly hydrogen throughout the whole 60 min measurement time as seen in Figure 3a,d. Despite the regeneration of the surface in UHV between the CO_2_RR runs, the ongoing usage of the same single crystal altered the surface. In the ∼third CO_2_RR measurement (Figure 3b,e), the surface exhibits HCPD within the first 15 min. However, the HCPD is only stable over a short time period, and with ongoing measurement time, the hydrocarbon selectivity decreases. In the ∼sixth CO_2_RR, the surface produces hydrocarbons over 1 h of CO_2_RR (Figure 3c,f). Further usage of the same single crystal in the CO_2_RR does not lead to an indefinite increase of hydrocarbon products. As soon as the HCPD is obtained on the surface, further usage of the same crystal does not increase the amount of produced hydrocarbons. Furthermore, the initial HCPD selectivity cannot be sustained for several hours, despite the still rough nature of this surface. This indicates that additional processes must be considered, as, for example, the possible time-dependent depletion of oxygen dissolved in copper, as previously suggested by Liu et al.^11^

**Figure 3:**
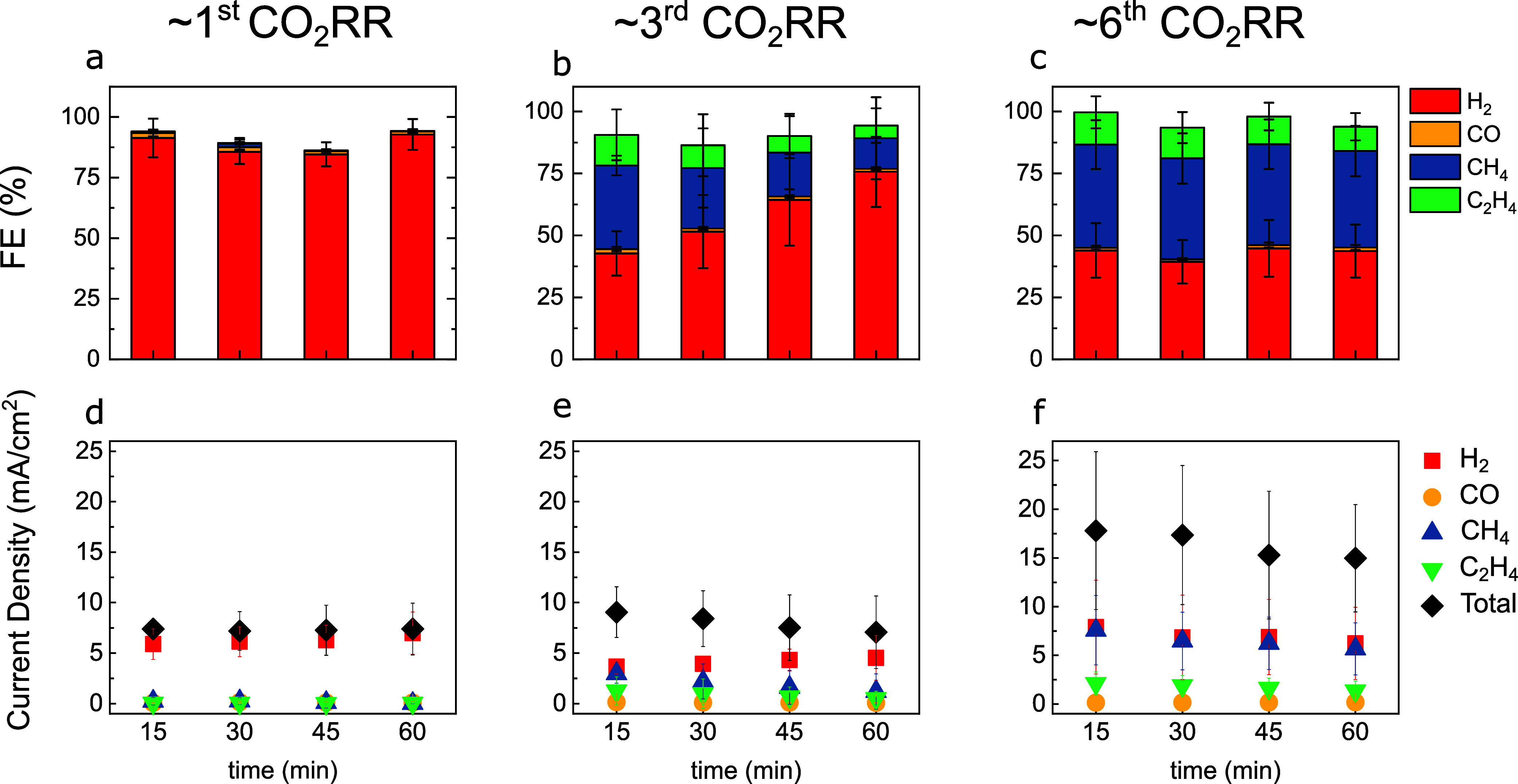
Selectivity and activity change of pristine UHV-prepared Cu(111) surfaces in dependence of the number of CO_2_RR runs. FE (a–c) and respective geometric partial current densities (d−f) over 1 h of CO_2_RR at −1.1 V vs RHE in CO_2_-saturated 0.1 M KHCO_3_, where gaseous products are sampled every 15 min. Between each CO_2_RR measurement (e.g., from a,d to b,e to c,f), the surface is reprepared in UHV. However, it should be noted that the morphological changes that the surface undergoes after each CO_2_RR are irreversible and that the subsequent UHV sputter/anneal cycle cannot restore the flat pristine Cu(111) surface.

Long-term CO_2_RR measurements have been conducted on both an H_2_PD and an HCPD Cu(111) surface for 18 h to investigate the stability of the catalytic activity and selectivity as shown in Figure 4. For the H_2_PD surface, the selectivity toward hydrogen stays the same for the whole measurement time. For the HCPD surface, the hydrocarbon production lasts primarily for the first hour of the CO_2_RR, before the surface starts to mainly produce hydrogen for the rest of the measurement time. Liu et. al reported a similar behavior for long-term CO_2_RR on polycrystalline Cu, and the time-dependent depletion of subsurface oxygen was discussed to be the main reason for the observed switch in the catalytic selectivity.^11^ Our data suggest that the structure of the Cu surface strongly influences its selectivity; the role of subsurface species such as oxygen can also not be neglected. In the course of the CO_2_RR, such species might be pulled out from the subsurface by reactants or intermediates (e.g., CO), giving rise to the roughening of the Cu single-crystal surface, together with a modification of its electronic properties. Thus, we hypothesize that even under potentiostatic CO_2_RR conditions, a dynamic redox behavior is observed under CO_2_RR with a strongly reducing applied potential, as long as there is oxygen dissolved in the near surface regions of Cu. Moreover, the microenvironment of the Cu surface and coverage of the different reactants and intermediates is expected to affect the surface and subsurface coverage of the oxygen species and thus also the material’s selectivity.

**Figure 4:**
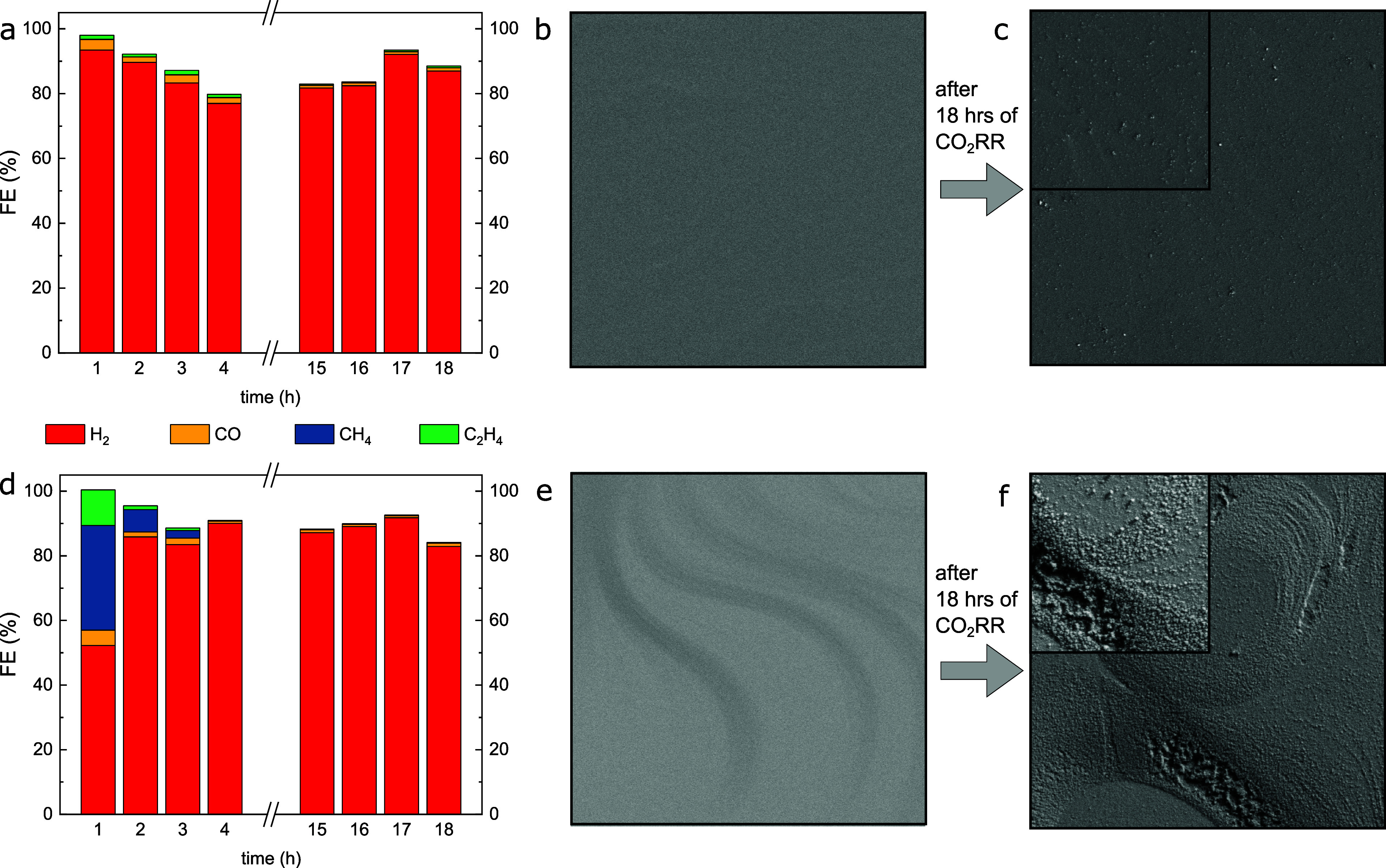
Faradaic efficiencies for long-term CO_2_RR for 18 h at −1.1 V vs RHE in 0.1 M KHCO_3_ for (a) H_2_PD UHV-prepared Cu(111) and (b) HCPD UHV-prepared Cu(111). SEM images of the H_2_PD surface (b) before and (c) after CO_2_RR and of the HCPD surface (e) before and (f) after CO_2_RR. Imaging conditions: (b, c, e, f) 15 μm × 15 μm, inset 4 μm × 4 μm, *V*_ac_ = 5 kV, *I*_beam_ = 0.2 nA.

All of our single-crystal surfaces are equally long exposed to air before being inserted into the electrolyte. Thus, for all, the native Cu oxides grown should be comparably thick. It is expected that the native oxide is reduced within the first few moments of CO_2_RR to metallic copper.^40^ The difference in selectivity can therefore not be assigned to different oxide thickness. Nonetheless, we cannot rule out that during each CO_2_RR cycle and subsequent air exposure, we get either O or C impurities into the crystal (outside the AES sensitivity), whose content might change as a function of the reaction time. The latter could affect the selectivity that we record. Nonetheless, structural changes might play a determining role here.

Overall, the shift in selectivity from an H_2_PD to an HCPD surface after multiple uses of the same individual crystal is reproducible across multiple different single crystals with the same surface orientation as those presented in this study. The same trend is not exclusive for Cu(111) but holds true also for different crystal facets as seen on UHV-prepared Cu(100) in Figure S3.

In order to further understand the differences in surface structure and to find the cause for the selectivity changes, we used microscopy techniques such as Scanning Tunneling Microscopy (STM) and Scanning Electron Microscopy (SEM). STM was applied to probe the atomic order and nanoscale features. Figure 5a,d shows atomic resolution as well as the Fourier transformed image of both surfaces, which is in agreement with the LEED pattern from Figure 1a,b. The hexagonal order of the atoms is well-displayed. Moving to a larger probe window as in Figure 5b,e (left), we can find for both surfaces well-defined terraces with monatomic steps (see Figure S4). Judging from these local STM images, both H_2_PD and HCPD surfaces look similar and cannot be distinguished from each other. However, by probing more spots on the HCPD surface, we are able to find major differences between both surfaces. Depending on the local probe area, the STM reveals regions on the HCPD surface displaying many step bunches (Figure 5e (right)), where most of them are of monatomic nature and few are multiatomic steps (see Figure S4).

**Figure 5:**
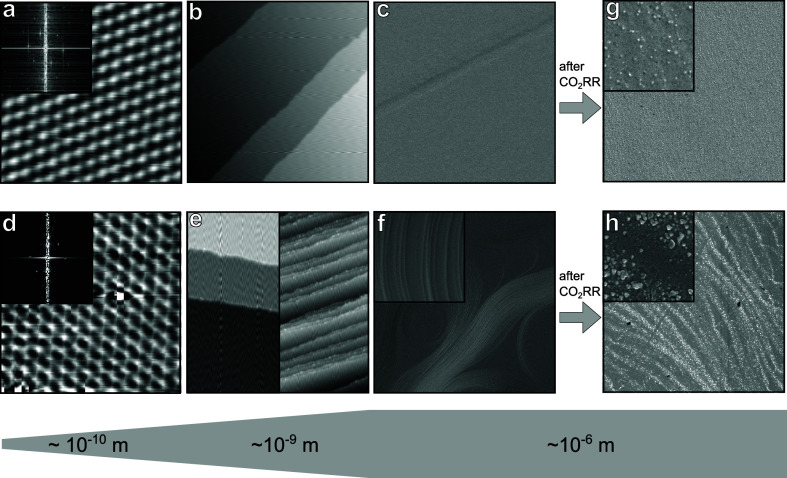
Microscopy images of the as-prepared H_2_PD (a–c) and HCPD (d–f) Cu(111) surfaces. STM images show atomic resolution and its Fourier transformed (a, d) and nanoscale (b, e) features. SEM images show the mesoscopic structure of both surfaces (c, f) as prepared and (g, h) after CO_2_RR. Imaging conditions: (a) 3 nm × 3 nm, *V*_s_ = 20 mV, *I*_t_ = 2 nA, (d) 3 nm × 3 nm, *V*_s_ = −5 mV, *I*_t_ = 5 nA, (b) 120 nm × 120 nm, *V*_s_ = 800 mV, *I*_t_ = 0.3 nA, (e) left 60 nm × 120 nm, *V*_s_ = 900 mV, *I*_t_ = 0.02 nA, (e) right 60 nm × 120 nm, *V*_s_ = 1000 mV, *I*_t_ = 0.06 nA, (c, f–h) 15 μm × 15 μm, *V*_ac_ = 5 kV, *I*_beam_ = 0.2 nA, inset 1 μm × 1 μm.

Thus, in order to get a holistic overview about the differences in surface structure, we use SEM to image the structure on a much wider range, namely, the mesoscopic scale. Figure 5c shows a pristine UHV-prepared Cu(111) surface under SEM. The surface is perfectly flat, and no features are detected under SEM other than widely spaced straight step edges. Figure 5g shows the morphology change of the surface after first CO_2_RR on the same single crystal. The overall surface is slightly roughened, and particles of size <50 nm are observed across the surface. Such particles likely arise from redox cycles underwent by a dynamic copper surface when transitioning from open-circuit potential conditions, where CuO_*x*_ species are present, to −1.1 V vs RHE and back in the presence of the CO_2_RR intermediates. Recently, Amirbeigiarab et al. observed the development of similar Cu nanocrystallites on Cu(100) during CO_2_RR conditions by *in situ* Scanning Tunnelling Microscopy.^41^ The exact chemical nature of the nanoparticles is difficult to ascertain, as AES only detected carbon, potassium, and oxygen in addition to Cu after CO_2_RR. Potassium is a leftover from the electrolyte, and adventitious carbon and oxygen arise from the electrochemical treatment and sample exposure to air after reaction.

Figure 5f shows a UHV-prepared crystal surface that has dramatically changed its mesoscopic structure after ∼6 cycles of alternating CO_2_RR and UHV treatments. The electrode exhibits a wavy structure across the entire surface. The inset of 1 μm × 1 μm shows a close-up of the wavy structure that consists of many steps. A full microscopic overview of the HCPD surface at different length scales can be seen in Figure S5. In STM, the step edges appear straight due to the narrow probe window, whereas at larger scales, SEM is able to reveal the curved nature of the step edges. Thus, the SEM images confirm that the HCPD surface has shorter terraces and displays a high density of steps, which is in agreement with LEED and CV scans. Overall, the surface looks clean and free from contamination, which is in agreement with the AES measurements. Figure 5h displays the same surface after the CO_2_RR. The wavy surface structure is still visible and decorated with particles. In comparison to the H_2_PD surface after the CO_2_RR in Figure 5g, the particles are no longer homogeneously distributed over the surface but clearly accumulated at the wavy steps, where the highest density of low-coordinated atoms is expected.

The SEM images also give a hint on how the wavelike structure evolves over time. The SEM image taken after CO_2_RR (Figure 5g) shows that the formerly flat surface is slightly structured, probably from CO-induced restructuring processes taking place during CO_2_RR.^41^ Subsequent UHV treatment and CO_2_RR cycles appear to promote these wavy structures. It is still unclear what exactly causes the unusual wavy shape of the step edges. Trace amounts of (sub)surface C, O, or K that are below the detection limit of AES could possibly stabilize the shape by pinning the step edges, although we could not yet detect any of these impurities on the UHV-regenerated (pre-exposed to CO_2_RR) as-prepared surfaces. However, we should mention that the structures we observe on the Cu(111) surface after extended operation, including the holes formed (Figure 4f), are similar to those characteristic of a Cu surface that underwent oxidative–reductive redox cycling. In the aqueous electrolyte under the OCP, the Cu surface is promptly oxidized, and during the CO_2_RR at negative applied potential and in the presence of surface reaction intermediates such as CO, dynamic oxidation–reduction processes are expected until all available near-surface oxygen has been pulled out, which we believe is the point where we see a selectivity switch back to hydrogen production. Thus, although the initial sample morphology is key to understanding the obtained selectivity, additional factors such as the presence of oxygen impurities and their temporal evolution during CO_2_RR must also be taken into account in order to explain the increase in the H_2_ production during extended operation.

The structural surface change between an H_2_PD and HCPD is subtle and difficult to observe based on ensemble-averaging diffraction methods, such as LEED or local atomic-scale methods, such as STM. Only the analysis on a mesoscopic scale, such as SEM, can reveal the major differences existing in the surface morphology.

Linking the surface morphology to the electrochemical measurements, we learn from Figure 2b that the ECSA-normalized partial current density for H_2_ is for both H_2_PD and HCPD UHV-prepared Cu(111) the same within the error, whereas the ECSA-normalized partial current density for both methane and ethylene has significantly increased for the HCPD sample. Thus, the same amount of generated H_2_ contributes differently to the total FE of both surfaces. Whereas the same amount of H_2_ contributes with 88% to the total FE of the H_2_PD, it contributes only to 40% to the total FE of the HCPD due to the additional amount of generated hydrocarbons (53%). This is assigned to the fact that in both samples most of the surface is flat (terraces) and thus inactive for CO_2_RR, favoring instead HER. Only the steps which take up a low fraction of the overall sample surface in both samples are active. Since the step density is significantly higher in the HCPD sample, the surface produces hydrocarbons.

Furthermore, the UHV treatment is mild enough that it preserves the terrace structure of the surface compared with harsher treatments like electropolishing and plasma etching used in our previous work that leads to a destruction of the terraces.^23^ Therefore, these well-defined clean surfaces enable the detection of Cu(110) surface features on the HCPD surface through CV curves (Figure 1f), hinting that these (110) sites are probably linked to the CO_2_RR active sites that initially lead to hydrocarbon production. A rough estimate of the total step edge length on both H_2_PD and HCPD surfaces extracted from STM images shows that a higher density of steps on the HCPD has also associated an increase in the ECSA as well as a decrease of the OH^–^ peak area (see Figure S6 for calculation).

The wavy structures on an HCPD surface consist of a large amount of steps, and it has been described that the oxygen uptake on Cu(111) is the highest for a high density of steps.^42^ The wave-like structures therefore oxidize the most when being exposed to air. Once the crystal is exposed to reducing conditions, the step bunches experience a surface reconstruction different from that of the prior perfectly flat terraces. As reported in the literature, the surface restructuring upon reduction prompts a rough surface with more uncoordinated sites.^40^ It was previously reported that step edges and under-coordinated sites can promote C–C coupling.^19,26,43^ Recently, Gauthier et al. conducted a detailed theoretical study on the roughening effect on oxide-derived Cu surfaces.^28^ They found that the roughening of oxide-derived Cu surfaces leads to the exposure of a broad variety of surface sites that cannot be found on pristine single-crystal surfaces. In agreement with their findings, we hypothesize that the surface modification of the wavy structures is a necessary condition to create active sites for CO_2_RR during electrolyses that do not exist for pristine atomically well-ordered crystals. We believe that along the curved step edges, it is likely that kinks and under-coordinated sites are found, leading to the observed hydrocarbon product distribution.

We present a multiscale study on UHV-prepared Cu(111) surfaces that spans atomic to mesoscopic characterization with microscopy, spectroscopy, and electron diffraction techniques. Although we are successful at restoring the local atomic order after each CO_2_RR cycle via UHV preparation methods, we find that transformations on the local nanoscale and mesoscopic structure take place after each reaction cycle and result in significant selectivity changes in CO_2_RR.

We learned from this study, in combination with our prior work,^23^ that flat surfaces (irrespective of whether they are Cu(100) or Cu(111)) favor the production of H_2_. Flat terraces contain only sites that are able to carry HER. The prior work hinted that roughening the Cu electrodes via chemical etching resulted in the production of hydrocarbon products. However, the etching has resulted in large structural changes, making it challenging to determine the exact C_2+_-product driving surface feature. In order to close this gap of knowledge, this work focuses exclusively on UHV-prepared Cu surfaces. Specific minimal surface changes were very carefully introduced on initially long-range-ordered surfaces to further trace down the crucial surface features that are relevant to tune the product selectivity from hydrogen toward hydrocarbons. Our new findings unveil that 110 structures are present on the Cu(111) single-crystal surfaces when hydrocarbons are produced.

Upon introducing mesoscopic wave structures on a formerly perfectly flat surface, we can attribute the observed selectivity changes to the irregular wave-like stepped structures formed after subsequent CO_2_RR cycles. It is astonishing that the majority of the flat atomically ordered Cu surface is inactive for CO_2_RR and that only a small fraction of the surface, which in this case we could identify as Cu(110) surface features in the CVs, is able to convert CO_2_ into hydrocarbons. Our wavy Cu surface consists of an increased amount of irregular steps with different orientations and exposes a large variety of surface sites that would not be exposed on perfectly flat crystals. Among these surface sites are special active sites (highly undercoordinated) driving the CO_2_RR. Our results highlight the important role of particularly oriented step edges for CO_2_RR. Therefore, further work should be directed toward elucidating the exact chemical and structural nature of these wavy step edges.

Besides featuring the key role of step edges, this work also demonstrates the importance of the pretreatment history of the Cu single crystal. The ongoing usage of the same single crystal strongly affects its CO_2_RR activity and selectivity. This is of key importance in the field since it reveals that work from different laboratories can be compared only if the state of the single crystal is pristine in all cases. Any subsequent use or additional CO_2_RR cycle will introduce irreversible morphological changes and, very likely, the incorporation of subsurface impurities during CO_2_RR that lead to distinct product selectivities. As illustrated in Figure 4 the role of impurities such as oxygen dissolved in the Cu crystals becomes more evident while monitoring the reaction selectivity over extended periods of time, when near-surface oxygen species might be successively depleted under the CO_2_RR microenvironment and applied reductive potential.

## Supporting Information Available

Additional experimental details, single-crystal preparation and methods, additional electrochemical results such as CO_2_RR on H_2_PD Cu(111) at different potentials, ECSA calculation, and CO_2_RR on Cu(100), additional STM and SEM images, estimation of step edge length and correlation to electrochemical properties (PDF)
