# Supplementary material for: The Influence of Mesoscopic Surface Structure on the Electrocatalytic Selectivity of CO2 Reduction with UHV-Prepared Cu(111) Single Crystals
Source: ACS Energy Lett. 2024 Jan 29;9(2):644–52. doi: 10.1021/acsenergylett.3c02693 (PMC10863400; doi:10.1021/acsenergylett.3c02693)
Supplement: Supplementary file 1 — nz3c02693_si_001.pdf [file nz3c02693_si_001.pdf]

The Influence of Mesoscopic Surface Structure on the Electrocatalytic Selectivity of CO_2 Reduction with UHV-prepared Cu(111) Single Crystals

Khanh-Ly C. Nguyen†, Jared P. Bruce†‡, Aram Yoon†, Juan J. Navarro†, Fabian Scholten†, Felix Landwehr†, Clara Rettenmaier†, Markus Heyde†*, Beatriz Roldan Cuenya†*

*e-mail: heyde@fhi-berlin.mpg.de; roldan@fhi-berlin.mpg.de

†*Fritz Haber Institute of the Max Planck Society, Department of Interface Science, Faradayweg 4-6, Berlin 14195, Germany*

†‡*Present address: Department of Chemistry and Biochemistry, University of Nevada, Las Vegas, Las Vegas, Nevada, USA 89154*

Supporting Information

Experimental Details:

Single Crystal Preparation. Cu(111) single crystals were cut and mechanically polished in house with a diameter between 8-10 mm and a height of 2-3 mm. The crystals are prepared in UHV as follows. First the crystals are sputtered with Ar^+-ions at a voltage of 1700 V and current of 12 µA for 30 min. Each sputter cycle is followed by an annealing cycle at T = 650°C for 10 min. This is repeated until a sharp Cu(111) Low Energy Electron Diffraction (LEED) pattern is obtained. For a freshly mechanically polished crystal, these UHV sputter and annealing cycles are repeated ~30 times before obtaining a sharp LEED pattern. After that, in between CO_2RR, ~8-10 cycles were needed until obtaining a sharp LEED pattern. Four final preparation cycles are completed prior to the removal of the sample from UHV for the electrochemical measurements. These cycles are completed with milder sputtering conditions using a voltage of 700V and a current of ~ 8 µA, followed by annealing at 650°C for 10 min. The surface is finally checked with Auger Electron Spectroscopy (AES) for cleanliness. The structure is again confirmed with LEED. Once the Cu(111) single crystal was exposed to CO_2RR, recovering its pristine morphology is only possible after a new mechanical polishing procedure followed by ~30 cycles of UHV Ar-sputter and annealing, which leads to the desired sharp LEED pattern for pristine Cu(111).

Surface Characterization

Our UHV chamber is equipped with Low Energy Electron Diffraction (LEED), Auger Electron Spectroscopy (AES), a sputter gun and an electron beam heater for the treatment of the surface of our single crystal samples. The base pressure in the preparation chamber is < 1 x 10^{-9} mbar.

Low Energy Electron Diffraction. LEED (Omicron SpectaLEED Control Unit) was carried out with a filament current of 1.68 mA and a screen voltage of 6kV. The LEED unit includes a Helmholtz coil to correct for stray magnetic fields.

Auger Electron Spectroscopy. AES measurements were taken with an electron spectrometer (Model DESA 150, Staib Instruments GmbH, Lagenbach, Germany). The Auger spectra were taken from 40eV to 1000 eV with a step size of 1.5 eV with an electron energy of 2kV. The raw spectra were analysed in CasaXPS by taking the derivative using a 5-point Savitzky-Goray algorithm. The samples were well grounded, and no further charge correction was required.

Scanning Electron Microscopy. The microscopy work was conducted using a Thermo Scientific Apreo scanning electron microscope (SEM) with a high stability Schottky field emission gun and a Trinity Detection system. The electron imaging was done using an acceleration voltage of 5 kV and a beam current of 0.20 nA.

Scanning Tunneling Microscopy. The STM work was carried out in an UHV system with a base pressure below 2 x 10^{-10} mbar. All images were acquired at 5 K using a PtIr tip. STM images were taken at constant current.

Electrolysis. All details of the electrochemical set-up, including the custom-built sample holder are described elsewhere.[36] After UHV preparation, the Cu(111) crystal is mounted ex situ on an in-house fabricated single crystal sample holder prior to immersion in the CO_2-saturated bicarbonate solution. All electrochemical measurements were taken in a standard glass H-type cell. Selemion was used as a membrane material to separate the two compartments of the H-type cell. A leak-free Ag/AgCl electrode (LF-2-100 from Alvatek) serves as reference electrode and a platinum mesh (99.9% purity from MaTeck) is used as counter electrode. The potential control was done using a Gamry 1010E potentiostat and (current-interrupt) IR compensation. The electrolyte is 0.1M $KHCO_3$ (ACS reagent 99.7%, Sigma-Aldrich, pH = 6.8). The electrolyte is bubbled with CO_2 gas for at least 30 min at a flow rate of 20 ml/min. The flow rate was monitored throughout the reaction to ensure accurate calculation of the faradaic efficiency. The single crystal is then inserted and a potential of -1.1 V vs. RHE is applied. A gas chromatograph (GC, Agilent Technologies 7890B) with HayeSepQ and HayeSepR columns was connected to the cell. The gaseous products were detected every 15 minutes with a flame ionization detector (FID) and a thermal conductivity detector (TCD). The CO_2 reduction reaction is carried out for 60 min. After the reaction, the crystal is thoroughly rinsed with ultrapure water (MilliQ, 18.2 MΩcm) and transferred back to the UHV chamber. The crystal is then again UHV-prepared, until good LEED and STM images are acquired before the next CO_2RR cycle.

Cyclic Voltammetry. All cyclic voltammetry scans were performed in a quasi in-situ electrochemical cell directly attached to the UHV chamber. The electrochemical cell is constantly filled with a pressurized argon flow (p_{Ar} = 1.1 bar). The used electrolyte is 0.1 M NaOH (semiconductor grade, 99.99% trace metals basis, Sigma-Aldrich, pH = 13). The electrolyte is bubbled with Ar gas for at least 30 min. All CVs were measured at a scan rate of 50mV/s.

CO_2RR on H_2PD UHV-prepared Cu(111) at various potentials

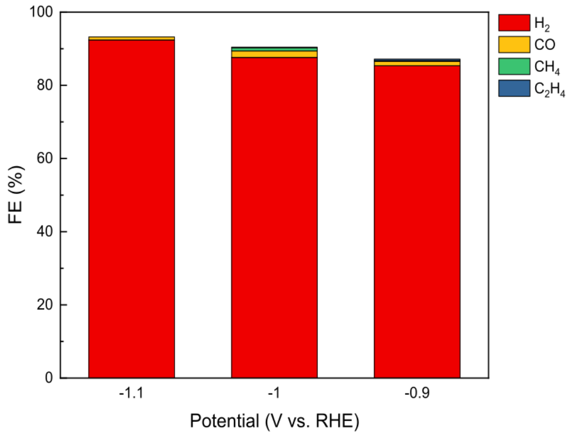

Figure S1: CO_2RR over UHV-prepared H_2PD Cu(111) measured at different potentials in 0.1 M $KHCO_3$ for 1 hour.

Electrochemical Surface Area Measurements

The electrochemical surface area (ECSA) was measured after 1h of CO_2RR at -1.1 V vs. RHE in 0.1M $KHCO_3$. The bubbling and the magnetic stirrer were stopped for the measurements. Cyclic voltammetry were taken at scan rates ranging from 100mV/s to 600mV/s.

The roughness factors are normalized to the measured capacitance of the atomically flat, pristine, H_2PD UHV-prepared Cu(111) surface.

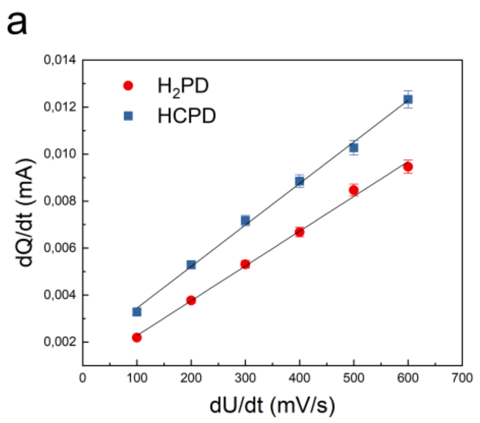

b

Cu(111) surface	Slope (A·s/V)	Roughness Factor
H_2PD	1.48E-5	1.00
HCPD	1.77E-5	1.19

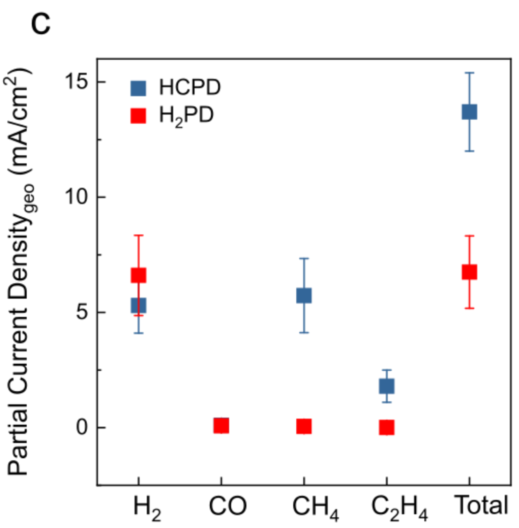

Figure S2: (a) ECSA measurements for the H_2PD and the HCPD Cu(111) surface. Data points are fitted by linear regression. (b) Slope of the linear fits are used to extract the roughness factors for H_2PD and HCPD Cu(111) surface. (c) Geometrical partial current densities for H_2PD and HCPD surface.

Selectivity changes during CO_2RR on UHV-prepared Cu(111) and Cu(100)

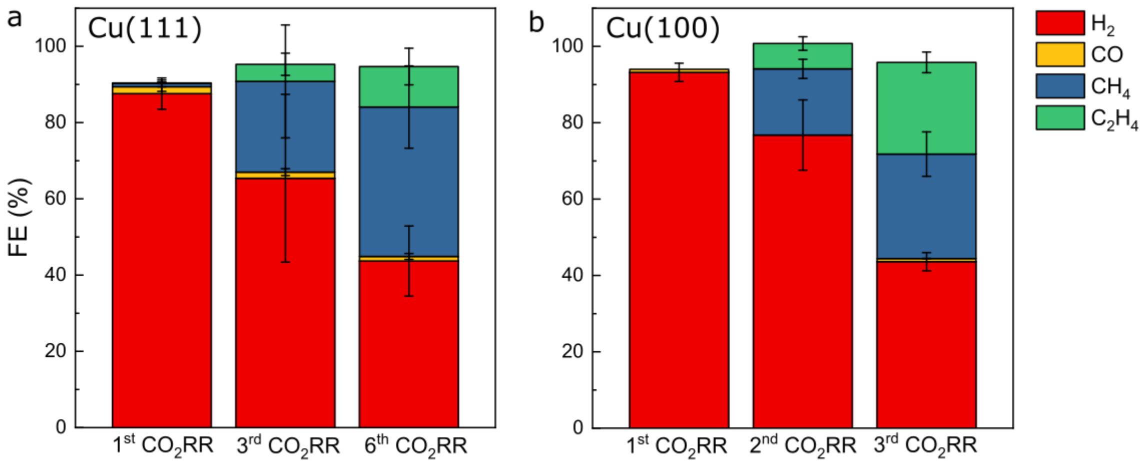

Figure S3: Selectivity changes in CO_2RR on UHV-prepared (a) Cu(111) at -1.1 V vs. RHE and (b) Cu(100) at -1.0 V vs. RHE in 0.1 M $KHCO_3$ in dependence of the history of the single crystal. The FE is averaged over a measurement time of 1 hr. In between each CO_2RR measurement, the crystals are re-prepared via sputtering/annealing in UHV.

The same trend of moving towards hydrocarbon production with the ongoing usage of the same single crystal electrode is observed for both, Cu(111) and Cu(100), which stresses the importance of taking into account the history of the single crystals. In the first CO_2RR measurement, mainly hydrogen is produced and with continuing usage of the same Cu(100) single crystal in CO_2RR and with UHV regeneration in between, the hydrocarbon production starts. Despite the UHV treatment (sputter/anneal) in between each CO_2RR, the surface morphology changes with continuing use. However, the change in selectivity happens earlier (with fewer CO_2RR cycles) for Cu(100) than for Cu(111), which is likely due to the higher intrinsic CO_2RR activity reported for Cu(100). Both Fig. 3 and Fig. S3 clearly state that with each cycle of UHV treatment and CO_2RR, we introduce changes to the surface that significantly impact the selectivity.

Scanning Tunneling Microscopy

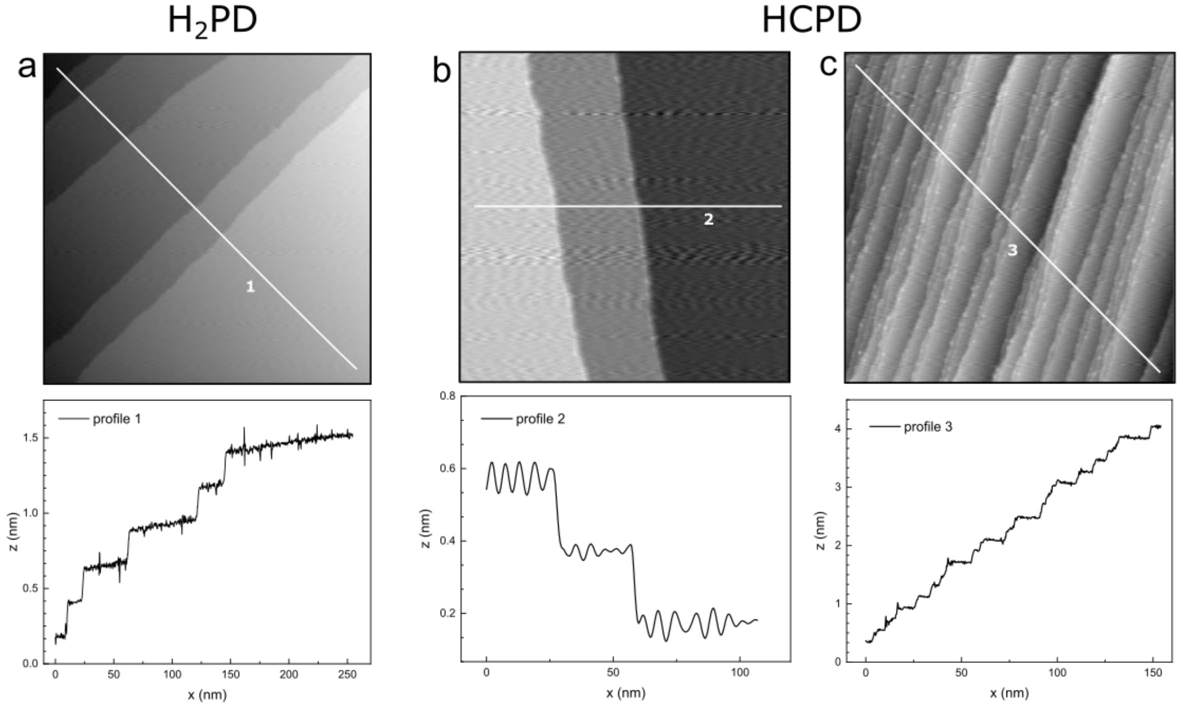

Figure S4: STM images and height profiles of the as-prepared (a) H_2PD Cu(111) surface and (b) and (c) of the as-prepared HCPD Cu(111) surface. Imaging conditions: (a) 200 nm x 200 nm, V_s = 800 mV, I_t = 0.3 nA, (b) 120 nm x 120 nm, V_s = 900 mV, I_t = 0.02 nA, (c) 120 nm x 120 nm, V_s = 1000 mV, I_t = 0.06 nA

Microscopic overview (SEM and STM) of the HCPD UHV-prepared Cu(111) surface

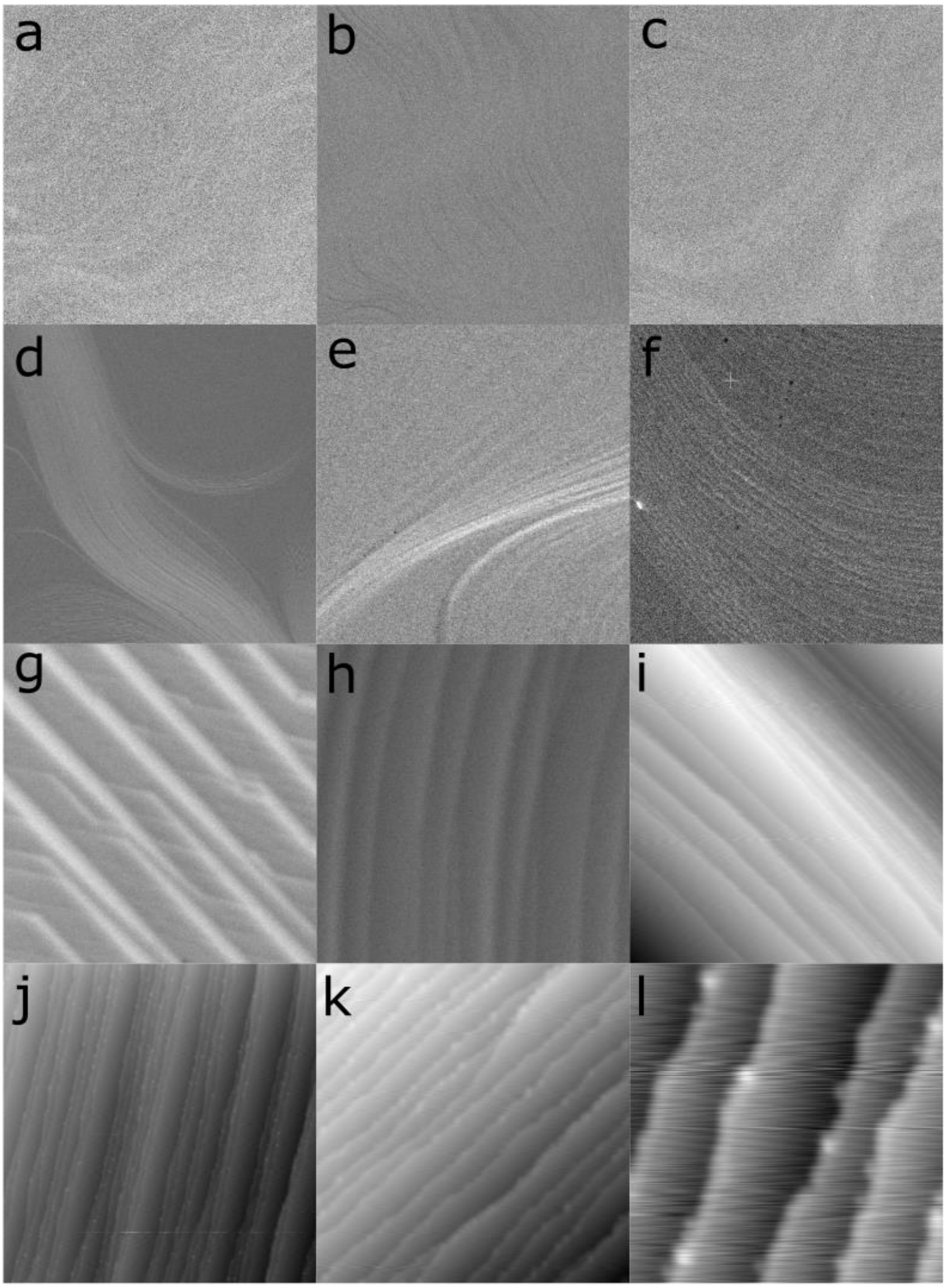

Figure S5: SEM images (a-h) and STM images (i-l) of the HCPD UHV-prepared Cu(111) surface, which were taken at different magnifications on different spots. This collection of images reveals the broad variety of surface features present on a HCPD UHV-prepared Cu(111) surface.

Imaging sizes: (a) 30 µm x 30 µm, (b) 20 µm x 20 µm, (c) 15 µm x 15 µm, (d) 10 µm x 10 µm, (e) 5 µm x 5 µm, (f) 3 µm x 3 µm, (g) 1 µm x 1 µm, (h) 500 nm x 500 nm, (i) 250 nm x 250 nm, (j) 130 nm x 130 nm, (k) 60 nm x 60 nm, (l) 20 nm x 20 nm,

Imaging conditions: All SEM images (a-h) were taken at V_{ac} = 5kV. For STM images (i) V_s = 780 mV, I_t = 0.13 nA, (j) V_s = 1000 mV, I_t = 0.05 nA, (k) V_s = 780 mV, I_t = 0.13 nA, (l) V_s = 500 mV, I_t = 0.09 nA

Estimation of step edge length on both H_2PD and HCPD Cu(111) and correlation to electrochemical properties

Hereby, we try to correlate atomistic properties such as the total step edge length with macroscopically measured electrochemical properties on both H_2PD and HCPD Cu(111).

The total step edge length was calculated based on STM images (e.g., see Fig. 5 b/e) and correlated with the ECSA (Fig. S2) and OH-peak area from the measured CV curves (see Fig. 1e).

In the following Fig. S6, we demonstrate on one example how the step edge length was calculated based on one specific STM image. The area of the single crystal is 0.2 cm².

The calculation was carried out on every obtained STM image and an arithmetic average value was calculated for both H_2PD and HCPD surfaces. In order to compare this structure-related atomistic property to electrochemical macroscopic properties such as the OH-adsorption peak area as well as the ECSA, a difference ratio was defined and calculated:

$$difference\ ratio = \frac{x(HCPD) - x(H2PD)}{x(H2PD)}$$

The difference ratio (Fig. S6c) reveals that the total step edge length increases by a factor ~23 from the H_2PD towards the HCPD surface, whereas the ECSA increases only by a factor of 0.19. Both properties show an increase from H_2PD to HCPD in contrast to the OH-peak area, which was found to decrease by a factor of ca. -0.28. The direction of the difference ratio value suggests that an increase in step edges is accompanied by an increase of the electrochemical surface area. This is in agreement with our observation that the HCPD surface consists of many step edges which results in a larger area exposed during electrochemical experiments, thus larger ECSA. In contrast, the OH peak area decreases with increasing step edge length. This is also in agreement with observations in literature, which described that OH adsorption rather takes place on the terraces. The absolute values of the difference ratios suggest that only a fraction of the step edges (~1 %) contribute to the macroscopic electrochemical properties.

We would like to stress that these estimations must be interpreted cautiously. The calculation of the length of the step edges is based on a small amount of STM images (<10 STM images for each surface). Taking into account the small sample size of data and the very local microscopic insight STM can offer, these calculations lack sufficient statistics and can thus only present a first analysis approach in order to gain insight into very complex structure-reactivity questions.

a

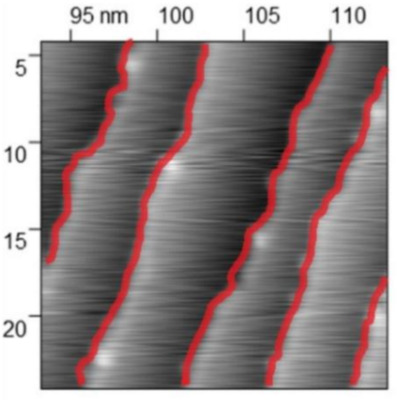

b

Image area [nm²]	Number of steps	Total step length [nm]	Step density [1/nm]	Step length on crystal [nm]
**20 x 20**	5	90	0.22	4.5 * E12
...	...	...	...	...

c

Property	H_2PD	HCPD	Difference ratio
**Step edges length [nm]**	1.05 * E11	2.49 * E12	23
**ECSA [a.u.]**	1	1.19	0.19
**OH peak area [AV]**	1.28 * E-6	9.21 * E-7	-0.28

Figure S6: (a) Example of measured step edge length on one specific STM image of the HCPD Cu(111) surface. (b) Table of one exemplary calculation of the total step length on the single crystal area of 0.2 cm² based on STM image (a). (c) Correlation between step edges length and electrochemical properties (ECSA and OH peak area) via a calculated difference ratio.
